# Higher mortality rates for large‐ and medium‐sized mammals on plantation roads compared to highways in Peninsular Malaysia

**DOI:** 10.1002/ece3.6827

**Published:** 2020-10-16

**Authors:** Jamaluddin Jamhuri, Mohd Anuar Edinoor, Norizah Kamarudin, Alex M. Lechner, Adham Ashton‐Butt, Badrul Azhar

**Affiliations:** ^1^ Department of Forest Science and Biodiversity Faculty of Forestry and Environment Universiti Putra Malaysia Selangor Malaysia; ^2^ Department of Wildlife and National Parks Kuala Lumpur Malaysia; ^3^ School of Environmental and Geographical Sciences University of Nottingham Malaysia Campus Semenyih Malaysia; ^4^ Lincoln Centre for Water and Planetary Health School of Geography University of Lincoln Lincoln UK; ^5^ British Trust for Ornithology Thetford UK; ^6^ School of Biological and Marine Sciences University of Hull Hull UK; ^7^ Biodiversity Unit Institute of Bioscience Universiti Putra Malaysia Serdang Malaysia

**Keywords:** forest, fragmentation, palm oil, road type, roadkill, wildlife

## Abstract

The fragmentation of forests by agricultural expansion, urbanization, and road networks is an ongoing global biodiversity crisis. In Southeast Asia and other tropical regions, wildlife populations are being isolated into pockets of natural habitat surrounded by road networks and monoculture plantations. Mortality from wildlife–vehicle collisions (WVCs) is contributing to a decline in many species of conservation priority in human‐modified landscapes. This study is the first in Malaysia to investigate factors affecting the occurrence of WVCs. We assessed roadkill data gathered by the Department of Wildlife and National Parks on small‐, medium‐, and large‐sized mammals in Peninsular Malaysia. We examined the relationship between wildlife road accidents and several environmental factors. We found a total of 605 roadkill animals, involving 21 species, which included three species classified as Endangered. Road type (plantation road or highway), year, and distance of the road from continuous and fragmented forests were significant in determining mammal mortality. Unexpectedly, the majority of road mortality occurred on palm oil plantation roads compared to highways. Mortality of small‐ and medium‐sized mammals was greater at locations further from continuous forest than those closer to fragmented forests. Segmentation of continuous forest by roads should be avoided wherever possible to reduce the threat of roads on crossing wildlife.

## Graphical abstract

Wildlife populations are being isolated into forest patches surrounded by roads. The majority of road mortality occurred on plantation roads compared to highways. Road type, altitude, and distance from forest were major drivers of mortality.

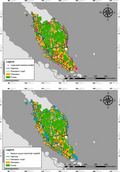

## 1 INTRODUCTION

Both vertebrate and invertebrate species occurring in forests are under increasing pressure from habitat loss, habitat fragmentation and hunting and/or illegal hunting, that is, poaching (Alroy, 2017; Jamhuri et al., 2018; Newbold et al., 2014; Samantha et al., 2020; Tee et al., 2018). Using satellite imagery, Curtis et al. (2018) reported that 27% of global forest loss (~5 million hectares of forest a year), over a fifteen year period (2001–2015), can be attributed to deforestation through permanent land use change for commodity production, such as palm oil, mining, oil and gas production extractive industries, and urban expansion.

Estimates of global forest loss from 2013 to 2014 are ~18.7 million hectares or a 9% decline in forest cover, partly due to the expansion of plantations and linear infrastructure including roads, railways, and power lines (Butler, 2015). These anthropogenic threats are common in tropical and subtropical countries with high biodiversity and diverse ecosystems (Alamgir et al., 2017; Laurance & Arrea, 2017). Road construction, in particular, creates a myriad of problems for wildlife such as forest degradation, barriers to access critical resources, such as food, shelter and breeding opportunities, increased accessibility to intact landscapes for hunters, and mortality from vehicle collisions (Alamgir et al., 2019; Ascensão et al., 2017; Chen & Koprowski, 2016; Jaeger & Fahrig, 2004; Laurance et al., 2009, 2014; Santos & Tabarelli, 2002). Wildlife–vehicle collisions (WVCs) are globally considered to be one of the major threats to wildlife (van der Ree et al., 2011). For example, in the United States, Loss et al. (2014) estimated that between 89 and 340 million birds are killed each year from collisions with vehicles.

Roads expose wildlife to vehicle collisions, resulting in an injury or a mortality to both wildlife and humans and can influence environmental and economic loss (Grilo et al., 2018). Often where road networks border forested areas, WVCs are high due to poor visibility for drivers (Kang et al., 2016). The vulnerability of wildlife to vehicle collisions is related to a range of factors such as mobility, habitat specificity, reproductive rate, resource need, and home range (Laurance et al., 2006). Furthermore, increased traffic volume, high speed roads and more highways, and wildlife–vehicle collisions (WVCs) have become more frequent, particularly in developing countries (Grilo et al., 2018). In Malaysia, increased deforestation and conversion of land to monoculture plantations as well as increased road expansion and development are likely to lead to increased WVCs as forests become more fragmented by plantation roads and highways (Azhar et al., 2013, 2014; Clements et al., 2014; Kolowski & Nielsen, 2008; Wadey et al., 2018).

**FIGURE 1:**
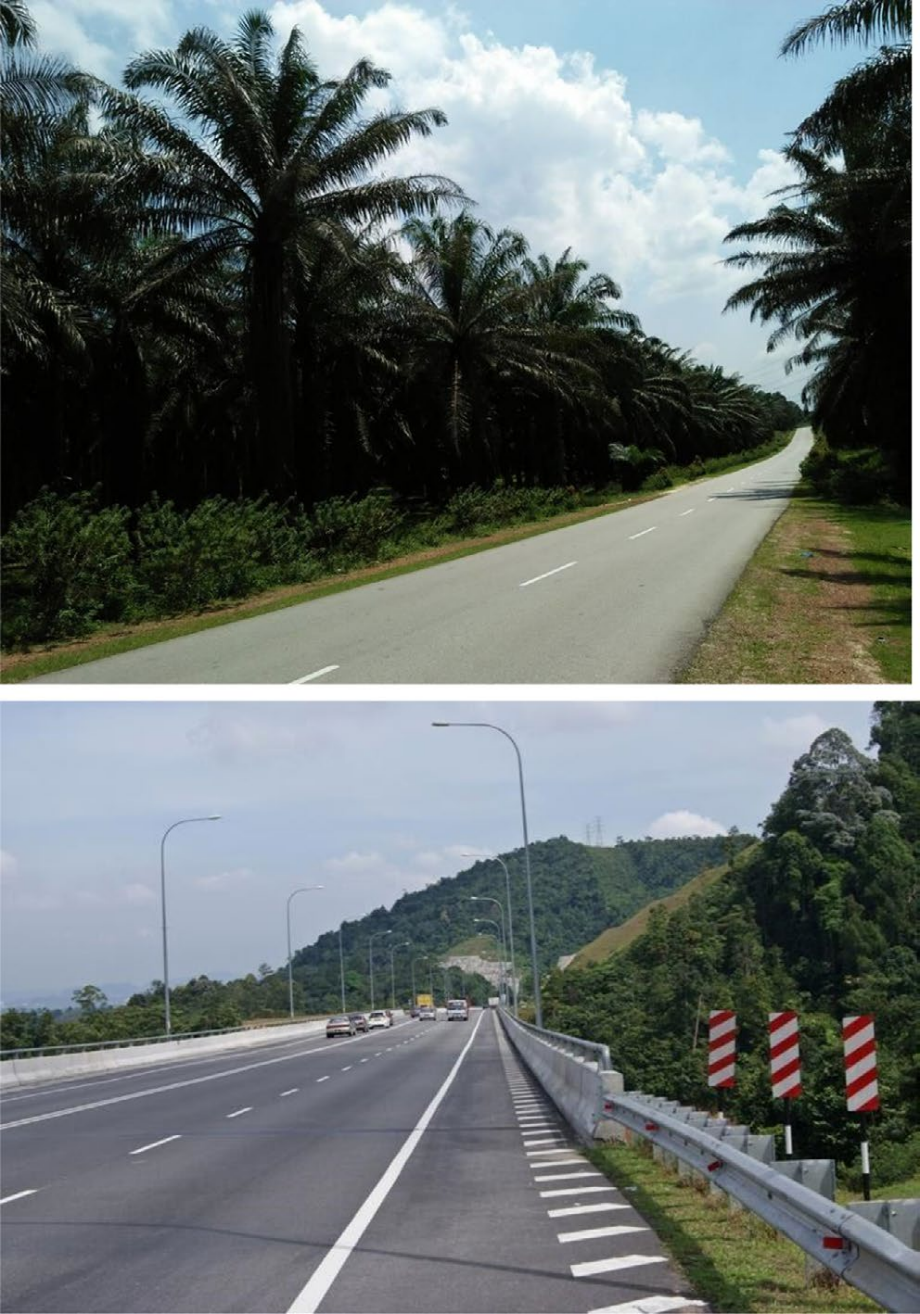
Typical agricultural plantation road (top) and highway (bottom) on Peninsular Malaysia

The aim of this study is to determine road‐related and landscape factors that influence the incidence of WVCs using wildlife roadkill occurrence data. We investigated whether WVCs were affected by the distance of the road (and road type) to continuous and fragmented native forests. We compared the effect of two paved road types (Figure 1): (a) highways with two or four‐lane roads and (b) plantation roads in palm oil production areas. We predicted that the WVCs would be greater in plantation roads due to their proximity to forest; as found in WVC studies in the United States, Brazil, and Italy (e.g., Eberhardt et al., 2013; Fabrizio et al., 2019; Grilo et al., 2018).

## 2 METHODS

### 2.1 Study area

The dataset for this study was collected from paved plantation roads and highways across Peninsular Malaysia (area of 130,598 km^2^; Figure 1) with more than 50% of its area ~150 m AMSL (range = 69.6–95.3 m). The average daily temperature is ~26°C (range = 21–32°C), with average rainfall/year ~2,400 mm (range = 1,800–3,000 mm per year). The natural habitat of Peninsular Malaysia is high in biodiversity, comprising mostly tropical rainforests due its location along the equator, including lowland/hill dipterocarp and peat swamp forests. These include Endangered tree species such as *Shorea pauciflora, Vatica kanthanensis*, and *Dipterocarpus sarawakensis* (Kochummen et al., 1990; Magintan et al., 2017; Okuda et al., 2003; Yule, 2010). There are 223 mammal species in Peninsular Malaysia. Of these, four species are classified as Critically Endangered, 13 are Endangered, 28 are Vulnerable, and 26 are Near Threatened (DWNP, 2017).

### 2.2 Data collection

Wildlife roadkill data were gathered over a five‐year periods (2010–2014) by Department of Wildlife and National Parks (DWNP) personnel in Peninsular Malaysia. Data were gathered by road patrols for specific regions (i.e., 47 DWNP district offices in 11 states). These teams comprised two personnel (with one being the driver and the other detecting roadkill), who both had expertise in identifying wildlife species, and in accordance with standard operation procedures established by the DWNP (DWNP, 2010). A mammal guide was also used (Francis, 2008) to further aid species identification. Patrols were conducted three times per week which encompassed most rural areas, with the vehicle driven at a constant speed (90 km/hr) on highways and plantation roads. We used distance patrolled by DWNP personnel from office to accident location as a measure of survey effort ($\bar{x}$ ± *SE* = 20 ± 0.60 km).

**FIGURE 2:**
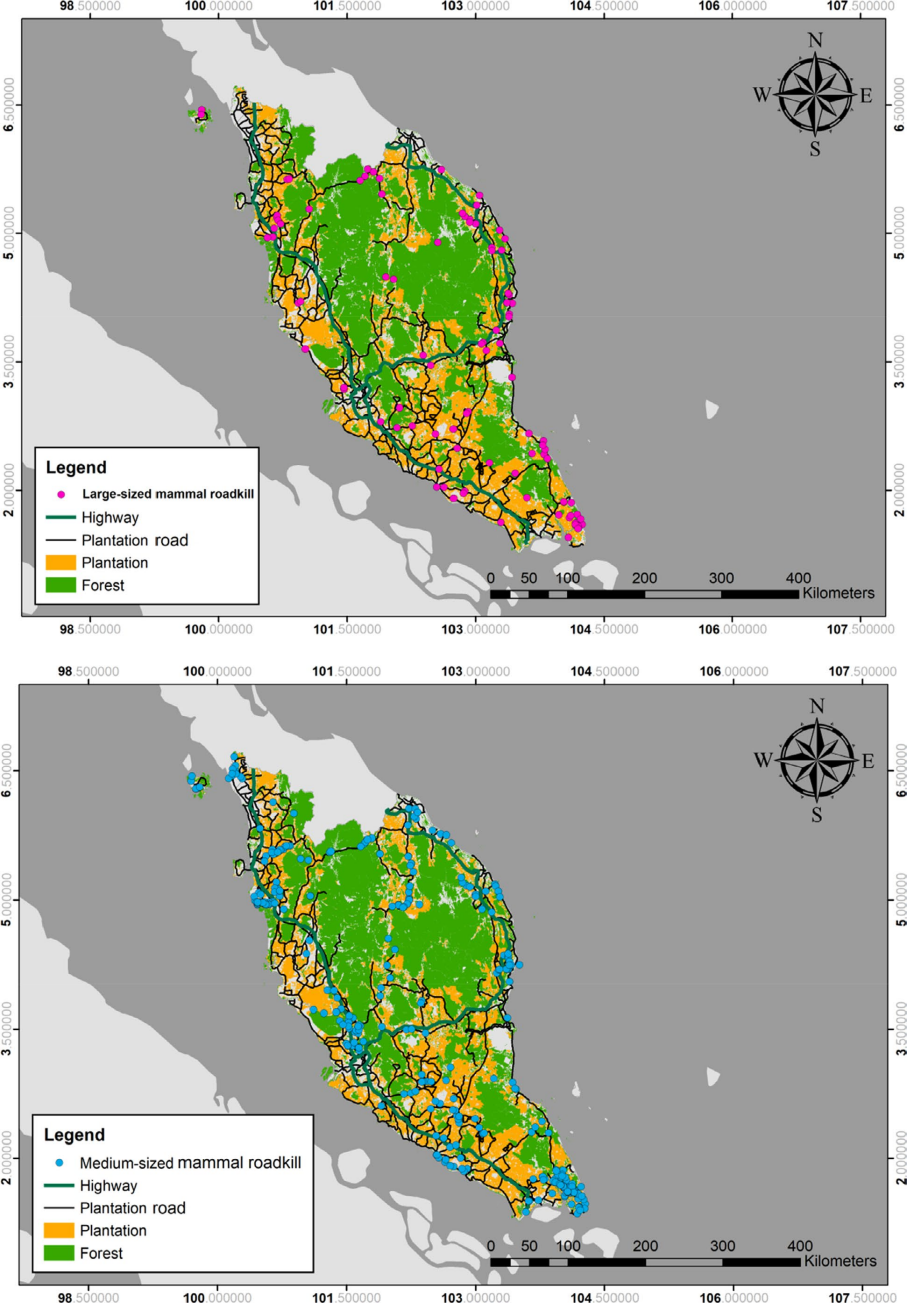
Locations of roadkills on Peninsular Malaysia (2010–2014)

Roadkill data were categorized according to typical body weight of the species into small‐ (<2 kg), medium‐ (2–15 kg), or large‐sized (>15 kg) mammals (Francis, 2008) (see Table 1). The personnel also recorded the road type (plantation road or highway) and roadkill location (Figure 2). To prevent illegal harvesting of animal body parts and organs and also to avoid recounts, carcasses were immediately transferred from accident location to the nearest DWNP office.

**TABLE 1:** List of small‐, medium‐, and large‐sized mammal roadkill species in Peninsular Malaysia (2010–2014)

Mammal type	Species		Family	Feeding guild	IUCN status	Number of roadkill	
	Common name	Scientific name				Highways	Plantation roads
Large mammals (>15 kg)	Wild pig	*Sus scrofa*	Suidae	Omnivore	LC	58	18
	Asian tapir	*Tapirus indicus*	Tapiridae	Herbivore	EN	21	7
	Asiatic wild dog	*Cuon alpinus*	Canidae	Carnivore	EN	1	0
	Asian elephant	*Elephas maximus*	Elephantidae	Herbivore	EN	1	0
Medium mammals (2–15 kg)	Common palm civet	*Paradoxurus hermaphrodites*	Viverridae	Omnivore	LC	138	79
	Long‐tailed macaque	*Macaca fascicularis*	Cercopithecidae	Omnivore	LC	148	7
	Leopard cat	*Prionailurus bengalensis*	Felidae	Carnivore	LC	28	23
	Dusky leaf monkey	*Trachypithecus obscurus*	Cercopithecidae	Herbivore	NT	14	0
	Malay civet	*Viverra tangalunga*	Viverridae	Omnivore	LC	3	11
	Smooth‐coated otter	*Lutrogale perspicillata*	Mustelidae	Carnivore	VU	7	7
	Malayan porcupine	*Hystrix brachyura*	Hystricidae	Omnivore	LC	4	7
	Pig‐tailed macaque	*Macaca nemestrina*	Cercopithecidae	Omnivore	VU	3	2
	Bearcat/ binturong	*Arctictis binturong*	Viverridae	Omnivore	VU	3	1
	Banded leaf monkey	*Presbytis femoralis*	Cercopithecidae	Herbivore	NT	2	1
	Yellow‐throated marten	*Martes flavigula*	Mustelidae	Carnivore	LC	2	0
	Javan mongoose	*Herpestes javanicus*	Herpestidae	Carnivore	LC	1	0
	Malayan pangolin	*Manis javanica*	Manidae	Carnivore	CR	1	0
	Large Indian civet	*Viverra zibetha*	Viverridae	Carnivore	NT	1	0
Small mammals (<2 kg)	Plantain squirrel	*Callosciurus notatus*	Sciuridae	Omnivore	LC	3	0
	Black giant squirrel	*Ratufa bicolor*	Sciuridae	Herbivore	NT	2	0
	Moonrat/gymnure	*Echinosorex gymnurus*	Erinaceidae	Carnivore	LC	1	0

Note

IUCN Status; IUCN Red List of Threatened Species: LC—Least Concern, VU—Vulnerable, NT—Near Threaten, CR—Critically Endangered.

### 2.3 Measurement of landscape characteristics

We measured the distances of road from the nearest continuous forest (an area more than 10,000 ha) and from the nearest fragmented forest (an area less than 10,000 ha) and altitude at the roadkill location using the measurement tool in Google Earth (GE) Pro (Version 6) (Table 2). GE Pro provided the remote sensing imagery for the measurement of the above variables. The GE images were acquired between 1 June and 4 September 2014 and commonly had a spatial resolution of 15 m per pixel (Potere, 2008).

**TABLE 2:** Summary statistics for distance between nearest continuous forest or fragmented forest and roadkill record location

Attribute	Mean	*SE*
Distances from the nearest continuous forest (km)	41.1	1.9
Distance from the nearest fragmented forest (km)	4.2	0.3

### 2.4 Data analysis

We used generalized linear mixed models (GLMMs) (Schall, 1991) to quantify relationships among roadkill and landscape‐level attributes (e.g., distance of road from fragmented forest), road type, and number of WVCs/year. Two additional models were developed to examine the effects of environmental variables on large‐ and medium‐sized mammal roadkill. We did not analyze small‐sized mammal roadkill due to insufficient sample size. We included five explanatory variables: distance of road from continuous forest (m), distance of road from fragmented forest (m), altitude (m), survey effort (m), road type (highways or plantation roads), and year. We used a Poisson distribution with a log‐link function in the modeling process. WVC location was used as random factor. The dispersion parameter was fixed at 1.

To examine the potential challenge arising from multi‐collinearity between predictor variables, we performed correlation tests among variables. No correlated variables were excluded as all variables were correlated below |*r*| = 0.7, the model distortion limit of Dormann et al. (2013). We fitted all possible regression models to select the final model (Schall, 1991). We followed Johnson and Omland (2004) by selecting data based on alternative hypotheses prior to data collection and analysis. We selected the most parsimonious models based on the minimum Akaike's information criterion (AIC) values (Anderson & Burnham, 2004). Since sample size (*n*), relative to the parameter (K), was large (*n*/*K* = 456/6 = 76 > 40) for at least one of the models, we did not use a corrected AIC (AICC) to compare the models (Anderson & Burnham, 2004). We reported the adjusted coefficient of regression, *r* ^2^ for the models. All analyses were performed in generalized linear models (GLMs) in GenStat 12th Edition (VSN International, Hemel Hempstead, UK).

## 3 RESULTS

### 3.1 General patterns of wildlife roadkill occurrences

During the five‐year period, the total survey effort was more than 9.1 million km (7.6 million km and 1.5 million km travelled on highways (*n* = 388 WVC locations) and plantation roads (*n* = 68 WVC locations), respectively). A total of 605 roadkill animals were recorded between 2010 and 2014 (Figure 2), with 106 large‐, 493 medium‐, and six small‐sized mammal records. In terms of feeding guild, carnivore, herbivore, and omnivore had 72, 48, and 485 records, respectively. Twenty‐one species were recorded, 11 of which are listed for protection under the Wildlife Conservation Act 2010 (Act 716). Three of the recorded species are listed as Endangered: the Malayan tapir (*Tapirus indicus*), Asian wild dog (*Cuon alpinus*), and Asian elephant (*Elephas maximus*). Three are listed as Vulnerable by the IUCN Red List (IUCN, 2017): the smooth‐coated otter (*Lutrogale perspicillata*), pig‐tailed macaque (*Macaca nemestrina*), and binturong (*Arctictis binturong*) (Table 1).

No small‐sized mammal roadkill was recorded on plantation roads, but six records were detected on highways. No roadkill was recorded involving the large‐sized carnivores in Peninsular Malaysia such as the Malayan tiger (*Panthera tigris*) and leopard (*P. pardus*). The common palm civet (*Paradoxurus hermaphrodites*) had the highest number of mortality records (Table 1). Based on the available data, the exact time of the WVCs was unknown and hence it was impossible to determine whether most accidents occurred during the day or night. However, we can assume that some roadkill animals were nocturnal (seven species or 33%). Most of the roadkill (12 species or 57%) were forest‐dependent species (Table 1), with the exception of wild pig, long‐tailed macaque, plantain squirrel, and common palm civet, which can be found in a wide range of habitats including forests and agricultural areas (Azhar et al., 2014; Tee et al., 2019).

Our data also indicated that solitary animals and common species were the most likely to have WVCs, whereas large‐sized conservation priority species such as the Asian elephant (*E. maximus*) and Asiatic wild dog (*C. alpinus*) were the least likely. However, the numbers of wildlife roadkill involving animals that occur in large groups and common species such as long‐tailed macaque (*Macaca fascicularis*), Common palm civet (*Paradoxurus hermaphrodites*), and wild pig (*Sus scrofa*) were moderately high.

### 3.2 Factors associated with mammal roadkill occurrences

The most parsimonious model for predicting total mammal roadkill was one in which road type and distance of road from continuous forest were the explanatory variables (Table 3). Our results revealed that the number of WVCs on the plantation roads was 0.42 times greater than the highways (Table 4). Plantation roads had higher WVCs irrespective of body size ($\bar{x}$ ± *SE* = 2.338 ± 0.326 roadkill/location) than highways ($\bar{x}$ ± *SE* = 1.149 ± 0.0317 roadkill/location). We found that the number of wildlife roadkill was higher in areas located further from continuous forests than fragmented ones (Table 4). Altitude, distance of road from fragmented forest, survey effort, and year had no significant effect on WVC occurrence. This model explained 28.14% of variation in wildlife roadkill across WVC locations (Table 3).

**TABLE 3:** Best subsets from candidate models

Response variable	Model	Explanatory variable	Adjusted *R* ^2^	AIC
Overall roadkill	1	Road type	20.40	205.81
	2	Model 1 + Distance from continuous forest	25.45	194.57
	3*	Model 2 + Year	28.14	194.17
	4	Model 3 + Distance from fragmented forest	28.77	194.19
	5	Model 4 + Altitude	28.94	195.38
	6	Model 5 + Survey effort	28.86	197.19
Large‐sized mammal roadkill	1	Year	2.07	342.91
	2	Model 1 + Road type	3.50	339.33
	3*	Model 2 + Distance from fragmented forest	4.56	336.99
	4	Model 3 + Distance from continuous forest	4.66	337.96
	5	Model 4 + Altitude	4.98	338.16
	6	Model 5 + Survey effort	4.92	339.65
Medium‐sized mammal roadkill	1	Road type	12.09	354.46
	2	Model 1 + Distance from continuous forest	15.42	342.47
	3	Model 2 + Year	19.04	333.21
	4*	Model 3 + Distance from fragmented forest	20.51	328.72
	5	Model 4 + Altitude	20.76	329.05
	6	Model 5 + Survey effort	20.63	330.85

Note

The most parsimonious model for each response variable (labeled with *) is determined based on a lowest AIC and complemented with an adjusted *R* ^2^.

**TABLE 4:** Coefficient of each explanatory variable in the most parsimonious models

Response variable	Explanatory variable	Slope	*SE*
Overall roadkill	*Road type*		
	Highways	0.000	
	Plantation road	0.424	
	*Distance of road from continuous forest*	3.620 × 10^–6^	1.287 × 10^–6^
	*Year*		
	2010	0.000	
	2011	−0.378	
	2012	−0.313	
	2013	−0.235	
	2014	−0.398	
Large‐sized mammal roadkill	*Road type*		
	Highways	0.000	
	Plantation road	0.175	
	*Distance of road from fragmented forest*	2.188 × 10^–5^	1.131 × 10^–5^
	*Year*		
	2010	0.000	
	2011	0.212	
	2012	0.733	
	2013	0.631	
	2014	0.755	
Medium‐sized mammal roadkill	*Road type*		
	Highways	0.000	
	Plantation road	0.454	
	*Distance of road from continuous forest*	5.225 × 10^–6^	1.501 × 10^–6^
	Distance of road from fragmented forest	−1.984 × 10–5	9.310 × 10^–6^
	Year		
	2010	0.000	
	2011	−0.462	
	2012	−0.469	
	2013	−0.364	
	2014	−0.640	

### 3.3 Roadkill occurrences involving large‐sized mammals

The most parsimonious model for large‐sized mammal roadkill was one that included road type, distance from fragmented forest, and year (Table 3). Our results showed that the number of WVCs involving large‐sized mammal on the plantation roads was 0.66 times greater than the highways (Table 4). Roadkill involving large‐sized mammals were more likely to happen on plantation roads ($\bar{x}$ ± *SE* = 0.368 ± 0.0861 roadkill/location) than highways ($\bar{x}$ ± *SE* = 0.209 ± 0.0222 roadkill/location). Our results also revealed that the number of large‐sized mammal roadkill was greater in areas located further away from forest fragments than those closer to the forests (Table 4). The number of large‐sized mammal roadkill was significantly influenced by year (Table 4). The lowest WVC occurrence ($\bar{x}$ ± *SE* = 0.118 ± 0.0446 roadkill/location) was in 2011, but the highest record ($\bar{x}$ ± *SE* = 0.310 ± 0.0511 roadkill/location) was in 2014. We did not detect significant effects from altitude, survey effort, and distance from continuous forest on the number of large‐sized mammal roadkill. The coefficient of determination, adjusted *R* ^2^ for this model, was 4.56% (Table 3).

### 3.4 Roadkill occurrences involving medium‐sized mammals

The most parsimonious model for explaining variations in medium‐sized mammal roadkill was one in which road type, distance of road from continuous forest, distance from fragmented forest, and year were the explanatory variables (Table 3). Our results showed that the number of roadkill involving medium‐sized mammals in the plantation roads was 0.4540 greater than the highways (Table 4). As with large‐sized mammal roadkill, more medium‐sized mammal roadkill were found on plantation roads ($\bar{x}$ ± *SE* = 1.971 ± 0.312 roadkill/location) than highways ($\bar{x}$ ± *SE* = 0.925 ± 0.0343 roadkill/location). The number of medium‐sized mammal roadkill was more likely to increase in areas located further away from continuous forests than those closer to the forests (Table 4). In contrast, the number of medium‐sized mammal roadkill decreased with distance from the nearest forest patch (Table 4). The number of medium‐sized mammals was significantly attributed to year (Table 4). The highest WVC occurrence ($\bar{x}$ ± *SE* = 1.633 ± 0.290 roadkill/ location) was in 2010 followed by 2013 ($\bar{x}$ ± *SE* = 1.147 ± 0.134 roadkill/location) then 2011 ($\bar{x}$ ± *SE* = 0.985 ± 0.0491 roadkill/ location) and 2012 ($\bar{x}$ ± *SE* = 0.958 ± 0.0572 roadkill/location) with the lowest record ($\bar{x}$ ± *SE* = 0.888 ± 0.0782 roadkill/location) in 2014. Finally, roadkill occurrences involving medium‐sized mammals were not influenced significantly by altitude and survey effort. This model explained 20.51% of the variation in medium‐sized mammal roadkill across WVC locations (Table 3).

## 4 DISCUSSION

This is the first study in Malaysia and perhaps Southeast Asia to quantitatively investigate factors affecting the occurrence of WVCs over larges scales. We showed that 28 Malayan tapirs, an Asian elephant, and a wild dog were killed in WVCs over the study period. The mortalities of these endangered species from WVCs will likely make a marked impact on their already declining populations due to anthropogenic threats such as logging, agricultural expansion, poaching, and urbanization (IUCN, 2017) and should cause national and international concern. It should be noted that our study only includes WVCs discovered by wildlife department personnel and that the populations of these species, in Peninsular Malaysia, have been declining based on the population data from IUCN (2017). Therefore, the impact of WVCs on Malayan Tapir and Asian Elephant populations is likely to be underestimated.

While carnivores are considered to be less common roadkill than mammals with other diet types (Ford & Fahrig, 2007), our results revealed otherwise, with carnivores being more common roadkill than herbivores. This is likely due to carnivores having bigger home ranges than herbivores in tropical rainforests, requiring them to travel greater distances and cross both highways and plantation roads (Linkie & Ridout, 2011; Naha et al., 2016; Ripple et al., 2014). Furthermore, our results revealed that the number of roadkill for medium‐sized mammals was higher compared than large‐sized mammals, likely due to larger numbers of medium‐sized mammals than large‐sized mammals, whereas the population number of small‐sized mammal species is probably larger than medium‐sized and large‐sized mammal, but detected less often, most likely due to the difficulty of detection as roadkill and the speed of patrol vehicle. Besides animal size, road type is another key factor that should be considered by wildlife agencies when implementing mitigation strategies or measures to reduce WVCs.

Our data can improve region‐wide conservation planning for identifying hotspot locations of wildlife road accidents in Peninsular Malaysia. However, there are limitations to our study. First, data were unavailable to indicate whether DWNP personnel invested differential survey efforts on highways compared with plantation roads. However, accumulated distances travelled by personnel on plantation roads and highway were not available to correct the analysis for survey effort. Second, our data describe the number of roadkill at each WVC location but not the number of roadkill/km/year. Third, the speed of the patrol vehicle (i.e., 90 km/hr) was likely too fast, resulting in some roadkill being undetected. And finally, the GE satellite imagery was acquired after data were collected, and therefore, landscape change during this survey period was not recorded.

### 4.1 Factors influencing wildlife road accidents

Our study found roadkill were higher on plantation roads compared to highways; a finding consistent with Orlowski and Nowak (2006) who suggested road‐related factors (e.g., road location and vehicle traffic volume) may influence roadkill patterns. The expansion of industrial‐scale plantations in Malaysia, particularly palm oil monoculture around forest reserves and protected areas, is likely to increase road construction (for transporting oil palm from plantation to mill) and subsequently cause more WVCs. Some mammal species such as primates and civets may utilize forest patches inside palm oil plantations for foraging (Azhar et al., 2013, 2014; Bernard et al., 2014) and thus are likely to cross roads to access these areas. Transient mammals utilizing palm oil habitat such as wild boar, tapir, and pig‐tailed macaque may cross roads in order to find food resources (Bernard et al., 2014; Sasidhran et al., 2016).

Forest‐dependent mammals will come into increasing contact with roads as their habitat is fragmented and destroyed leading to the likelihood of an increase in the relative number of mammals involved in WVCs. Plantation roads may also function in a similar way as other linear clearings (e.g., along roads, power lines, or train tracks) whereby there is greater abundance and richness in this edge habitat. Cerboncini et al. (2016) reported a slight increase in small mammal richness at forest‐railway edges in the Atlantic Forest of southern Brazil. Roadkill may also be higher on plantation roads than highways as plantation roads are generally narrower, with thicker vegetation at the road edge. Thicker roadside vegetation may muffle light and sound from vehicles causing wildlife to be unaware of the road presence (Goosem, 2007; Jaarsma et al., 2006; Siers et al., 2016).

Interestingly, the majority of roadkill occurred at higher altitude as oppose to low‐lying or coastal areas, possibly due to the higher abundance of forest mammals present at this altitude. Furthermore, the majority of lowland forest habitat in Peninsular Malaysia has already been developed to establish agricultural areas or human settlements (Miettinen & Liew, 2010).

Medium‐sized mammal roadkill were more likely to occur in areas furthest from continuous forest and near fragmented forest patches. This may be due to wildlife usually expanding their home range to forage and breed as their habitat is reduced and/or becomes more degraded. Furthermore, higher populations of herbivores in the forest patches are likely to be attributed to the lack of carnivores.

The number of WVCs varied year to year, which could be influenced by further deforestation and agricultural expansion. Malaysia lost 2.65 Mha (decreased by 17%) of humid primary forest from 2002 to 2019 (Global Forest Watch, 2020). Conversely, palm oil area has steadily increased from 5.74 Mha in 2016 to 5.90 Mha in 2019 (increased by 2.8%) (MPOB, 2020).

As palm oil plantations in Peninsular Malaysia expand in number and area, more new roads are being constructed that border intact forests and forest reserves. These areas are likely to result in increased roadkill ensuing roadkill hotspots, which require appropriate mitigation (Zimmermann Teixeira et al., 2017). Ultimately, a decline in wildlife will likely occur in biodiversity‐rich areas (Fa et al., 2005).

### 4.2 Management implications

Our study is the first to record mortality data and some of the variables that cause it. The study is a useful starting point for finding hotspots and guiding future mitigation. As the extent of road networks and the area of palm oil plantations are growing in biodiversity‐rich countries in Southeast Asia, it is imperative that wildlife protection agencies impose measures that minimize the ecological impacts this causes, including the impact of WVCs. We suggest that roadkill hotspots should be used as an indicator of the sites for mitigation, particularly in palm oil plantations.

## CONFLICT OF INTEREST

The authors declare that they have no conflict of interest.

## AUTHOR CONTRIBUTIONS

**Jamaluddin Jamhuri:** Conceptualization (equal); data curation (equal); formal analysis (equal); investigation (equal); methodology (equal); project administration (equal); software (equal); visualization (equal); writing – original draft (equal). **Mohd Anuar Edinoor:** Data curation (equal); investigation (equal); methodology (equal); resources (equal); writing – original draft (equal). **Norizah Kamarudin:** Conceptualization (equal); funding acquisition (equal); writing – original draft (equal); writing – review & editing (equal). **Alex M. Lechner:** Writing – review & editing (equal). **Adham Ashton‐Butt:** Writing – review & editing (equal). **Badrul Azhar:** Conceptualization (equal); data curation (equal); formal analysis (equal); investigation (equal); methodology (equal); project administration (equal); software (equal); supervision (equal); visualization (equal); writing – original draft (equal); writing – review & editing (equal).

## Data Availability

Empirical data have been archived in DataDryad: https://doi.org/10.5061/dryad.gtht76hh8.
